# Distance and Character-Based Evaluation of the V4 Region of the 18S rRNA Gene for the Identification of Diatoms (Bacillariophyceae)

**DOI:** 10.1371/journal.pone.0045664

**Published:** 2012-09-21

**Authors:** Ian A. Luddington, Irena Kaczmarska, Connie Lovejoy

**Affiliations:** 1 Department of Biology, Mount Allison University, Sackville, Canada; 2 Québec-Océan, Département de Biologie and Institut de Biologie Intégrative et des Systèmes, Université Laval, Québec, Canada; Instituto de Biologia, Brazil

## Abstract

DNA barcoding is a molecular tool that exploits a unique DNA sequence of a standardized gene or non-coding region for the species identification of unknown individuals. The investigation into a suitable barcode for diatoms is ongoing and there are several promising candidates including mitochondrial, plastidial and nuclear markers. We analyzed 272 sequences from 76 diatoms species in the orders Thalassiosirales, Lithodesmiales and Cymatosirales, using distance and character based approaches, to assess the applicability of a DNA barcode based on the hypervariable V4 region of the nuclear 18S rRNA gene. We show that the proposed V4 barcode separated ca. 97% of all centric diatom taxa tested using a threshold *p-*distance of 0.02 and that many problem pairs were further separated using a character based approach. The reliability of amplification, extensive reference library and variability seen in the V4 region make it the most promising candidate to date for a barcode marker for diatoms particularly when combined with DNA character analysis**.**

## Introduction

Diatoms are ubiquitous and morphological species are widely used to identify environmental changes in both freshwater and marine ecosystems [1]. Diatoms, especially the thalassiosiroids are also responsible for much of the primary production throughout the world oceans [2]. A rapid means of correct species identification is essential to fully exploit high throughput environmental sequence surveys, archived material, including ancient DNA, and data from clone libraries of uncultivated eukaryotes. Accurate identification of diatoms will also facilitate expanded monitoring efforts in the face of natural versus anthropogenic changes in marine and freshwater ecosystems. The high number of described morpho-species and an ever increasing number of newly discovered semi-cryptic and cryptic species adds to the challenges of expediency in the routine identification of diatoms. Additionally, in the last decade or so, nearly every study applying a combination of molecular and classical methods to taxonomically re-apprise “difficult” species resulted in discovery of species complexes [3], [4], [5], highlighting the need to revise interpretations of previous findings in microbial population and community ecology and geography.

In light of this challenge, molecular methods can add to the taxonomic tool-kit by finding a genetic distance between organisms and resolving species boundaries. DNA barcoding takes this idea in molecular taxonomy even further by proposing a standardized, short DNA fragment that can consistently recognize species over a wide range of organisms [6], thereby providing a common metric for populations of conspecifics worldwide and aiding in the identification of ambiguous species.

For animals, a fragment of the 5′ end of the mitochondrial gene encoding for the cytochrome c oxidase subunit 1 (*cox*1), is sufficient for the identification of approximately 96% of species in seven phyla [6]. Other groups, for example, cnidarians [7], land plants [8] and fungi [9] do not segregate well using this gene. The utility of *cox*1 to identify protists, which are phylogenetically diverse and have uncertain evolutionary relationships, is mixed. The *cox*1 marker and other barcodes tested in green algae are ineffective [10] due to inconsistent amplification. Among the major macroalgae *cox*1 is a useful marker for rhodophytes [11], while in some phaeophytes (specifically the genus *Alaria*) the gene fragment cannot discriminate well defined species [12]. The *cox*1 marker is even less promising for dinoflagellates [13], and other short DNA sequences such as the internal transcribed spacer (ITS) region of the gene coding for rRNA [14] or the mitochondrial cytochrome *b* gene [15] seem to be more promising.

Up until now, four markers have been evaluated systematically for diatoms; *cox*1 [16], [17], [18], the plastidial *rbc*L gene [19], [20], nuclear rDNA ITS region [16], [18], [21] and the nuclear small subunit rRNA gene (18S) [22]. Detailed discussions of the advantages and disadvantages of the first three markers are treated in the publications cited above and are therefore not repeated here. The 18S rRNA gene is a frequently used marker for deep phylogenetic research, but is relatively long and the utility of short variable regions within the 18S rRNA gene is more promising [22].

Because of its high and unrivaled amplification success and reasonable resolving power the potential utility of the 18S rRNA gene as a species specific barcode has been highlighted at least within one large dataset tested [22]. Although Moniz and Kaczmarska [18] found that a 1600 bp long fragment of the ca. 1800 bp 18S rRNA gene did not contain sufficient variability to delineate species, another approach [22] demonstrated that a shorter region of the gene could be used instead. Zimmermann *et al.* [22] systematically tested the entire 18S gene sequence and identified promising regions based on their variability. Following sequencing of 123 freshwater test-taxa, they found that the ca. 30 bp V4 region was the most variable and this and flanking regions (420 bp) discriminated all but eight species from 123 and they posited that it could serve as a barcode for diatoms. The eight morpho-species that were unresolved all belonged to the thalassiosiroid genus *Stephanodiscus* and included*: S. agassizensis, S. binderanus, S. hantzsxhii, S. minutulus, S. neoastraea, S. niagarae, S. reimeri,* and *S. yellowstonensis.* All eight showed very low divergence levels overall including in the V4 region. Therefore, the authors suggested that the resolving power of V4 may be limited to well diverged species, while in the closely related species complexes or groups including cryptic and recently evolved species it may be best combined with more sensitive markers, such as 5.8-ITS-2 [22]. Other recent research indicates that the V4 region more closely approximates the variability of the entire 18S gene compared to the V9 hyper-variable [23], another candidate barcode region [24] albeit not in diatoms. Since Zimmerman *et al.* [22] focused on mostly freshwater genera and the thalassiosiroids were among the least successfully resolved taxa and contained many similar morpho-species, the aim of the present study was to further investigate the utility of the V4 region of the 18S gene for species identification, particularly in this group. We focused on mostly marine, closely allied Thalassiosirales and select other diatoms that were not included in the earlier study. The development of a standardized DNA marker with a large reference database in this group would significantly aid our understanding of the ecology of the diatoms with special emphasis on the thalassiosiroids. Members of this order form the most important constituent of summer blooms in the North Atlantic. A timely and reliable method of species identification would enable refined analyses of bloom dynamics, taxonomic composition and the characterization of long-range transport and species invasions, all increasingly important in a rapidly changing environment. Not only common species and main bloom constituents but also members of the “rare biosphere” could be readily elucidated through the use of a V4 based barcode in next generation high throughput sequencing including 454 pyrosequencing technology [25].

Our approach was to amplify and sequence the V4 region from three marine, species rich orders: Thalassiosirales, Lithodesmiales and the little studied Cymatosirales. We combined the new sequences with sequences of the V4 region that were available from GenBank, which we curated with the objective to further test the resolving power of the 18S V4 region as a possible barcode for diatoms. We expanded the specific and geographic coverage used by Zimmermann *et al.* [22] for the V4 test-set and included biologically defined species and a greater number of closely related morpho-species. Additionally, we evaluated the recently advocated character based approach [26], [27] to identify diatom sequences from the V4 region when unresolved by distance methods alone.

## Materials and Methods

Overall we obtained 272 sequences (**Table S1**) from 272 strains, clones or isolates. DNA template for this study was extracted from 42 monoclonal cultures previously established in our Mount Allison lab, and 32 additional strains from the National Center for Marine Algae and Microbiota (NCMA). Monoclonal cultures from 10 sex compatible clones of *Tabularia fasiculata* (order Fragilariales) and 3 *Campylosira cymbelliformis* (Cymatosirales) were sequenced as part of the test set to represent biologically defined species. DNA was also extracted from 8 single chains, of known species, isolated from environmental samples. At Mount Allison, diatom cultures were grown and their DNA extracted as described by Moniz & Kaczmarska [21] and MacGillivary & Kaczmarska [20] for a total of 82 new sequences. Voucher SEM images of the clones and strains can be retrieved via the BOLD accession numbers listed in **Table S1**.

The remaining 190 sequences were retrieved from Genbank and in total 76 species were analyzed, 30 from this study and 46 from GenBank. These retrieved sequences included much of, or the entire 18S gene and were trimmed to cover only the V4 region and flanking regions. Sequences from GenBank were selected based on the availability of corroborative evidence of species identity from published sources or communication with the depositors.

The V4 region along with conserved flanking regions (approximately 420 bp) was amplified using primers D512: 5′-ATT CCA GCT CCA ATA GCG-3′ and D978: 5′-GAC TAC GAT GGT ATC TAA TC-3′ following Zimmermann *et al.* [22].

Reactions of 25 µL for all cultured strains contained 12.5 µL of GoTaq Mastermix (Promega), 0.75 µL each of forward and reverse primers (final concentration of 0.3 µM), 9 µL of DEPC treated water and 2 µL of DNA template. Cycling conditions following an initial denaturation step of 30s at 95°C were 35 cycles of 30s at 94°C, 30s at 50°C and 30s at 72°C. Strains which exhibited low PCR yield were subjected to a second round reamplification under the same conditions.

Single chains isolated directly from seawater or ethanol preserved samples were subjected to a two round, nested-PCR protocol following Lang & Kaczmarska [28] with the following modifications: First round primers were 18F [29] and ITS4 [30] and the cycling conditions were an initial 3 min at 94°C followed by 14 cycles of 94°C for 30s, 48°C for 30s and 72°C for 1 min and then 19 cycles of 94°C for 30s, 48°C for 30s and finished with 72°C for 2 min 30s (increased by 10s each round). The second, nested round used 1 µL of first round PCR product as template, and otherwise followed the same cycling conditions and reaction volumes as for cultures. PCR products were then visualized on a 1.2% agarose gel precast with SYBR safe gel stain (Invitrogen). Post-PCR SEM was performed on two isolates of *Thalassiosira anguste-lineata* following Lang & Kaczmarska [28].

PCR products were purified and sequenced at McGill University and Génome Quebec by Sanger sequencing (3730xl DNA analyzer, Applied Biosystems). Sanger sequencing was preferred because we were using direct PCR on morphologically identified cells and the relatively few samples were not compatible with high throughput sequencing. Additionally, the accuracy and ability to cross-check base-calling allowed us to produce robust non ambiguous reference sequences, which are required for environmental gene surveys. Resultant chromatograms and sequences were inspected, edited and checked against similar GenBank sequences using the NCBI Basic Local Alignment Search Tool (BLAST), further checked for correct base-calling using FinchTV [31] and the alignment was manually refined with BioEdit [32]. Our final sequences were 333 bp long after primer sequences and the redundant, super-conserved downstream region (totaling to 420 bp in Zimmermann *et al.* [22]) was removed. Sequence analysis including the calculation of *p*-distances was conducted using MEGA5 [33]. Uncorrected *p-*distances were chosen as other models tested did not give significantly different distances (i.e. K2P distances). The maximum-likelihood phylogenetic tree was constructed in MEGA5 using the Tamura-3-paramter model [34] based on the best fit subsitution model and which produced trees closest to previously resolved phylogenies [35]. Identical sequences were removed to improve terminal branch clarity.

Character-based analysis was performed manually by analyzing sequence motifs within the Sequence Data Explorer in MEGA5 [33] because the chosen groups contained a small number of variable sites and sequences and therefore software was not required. Pure diagnostic (i.e. a transition or transversion unique to one group of sequences or species) and compound private diagnostics (i.e. a combination of two substitutions which alone are not unique to a group of sequences or species yet together are) were discerned from sequence alignments following Sarkar *et al.* [36] and used to classify sequences based on a neighbor-joining (NJ) guide tree. Unlike Sarkar *et al.* [36] however, a doubly compound private character (i.e. three private characters at different variable sites) was used as a pure diagnostic for the classification of one sequence group (*S. costatum* Subgroup B and *S. grethae*). This analysis method was used for all *Skeletonema* species as well as all Cymatosirales species because these genera contained several poorly resolved morpho-species and were represented by multiple sequences per species.

## Results

### Amplification and Sequencing

We successfully amplified and sequenced 30 species from 17 genera. Amplification success for all clones was 100% for all cultured strains and 47% for single chains. All successful amplifications were sequenced. Some strains, including all of the Cymatosirales but also *Skeletonema marinoi*, and *S. menzelli* needed to be amplified a second time from the first PCR product to ensure sufficient DNA for sequencing. The alignment of sequences was not collinear with insertions and deletions (indels), especially within the V4 region. However, conserved flanking regions and species-specific sequence motifs allowed for unambiguous manual alignment. Two sequences, from *Extubocellulus cribriger* and *E*. *spinifer* (CCMP391 and 393) had an approximately 150 bp insertion downstream of the V4 region, however, since these indels were outside the region of interest they had no effect on alignment. Although these two species amplified and sequenced well for the V4 region, there were two bands visualized in the gels used to verify the amplification steps with one of the bands 450 bp, which was the target length and the second band approximately 600 bp. PCR products were sequenced directly without excision and the longer of these products sequenced preferentially. Likewise, similar double bands were seen for *Minutocellus polymorphus* (CCMP499*)* though in this case the smaller band was stronger and sequenced preferentially. After trimming, final aligned sequences containing the ca. 30 bp highly variable V4 region were 333 bp in length.

### Distance Analysis

**Figure 1:**
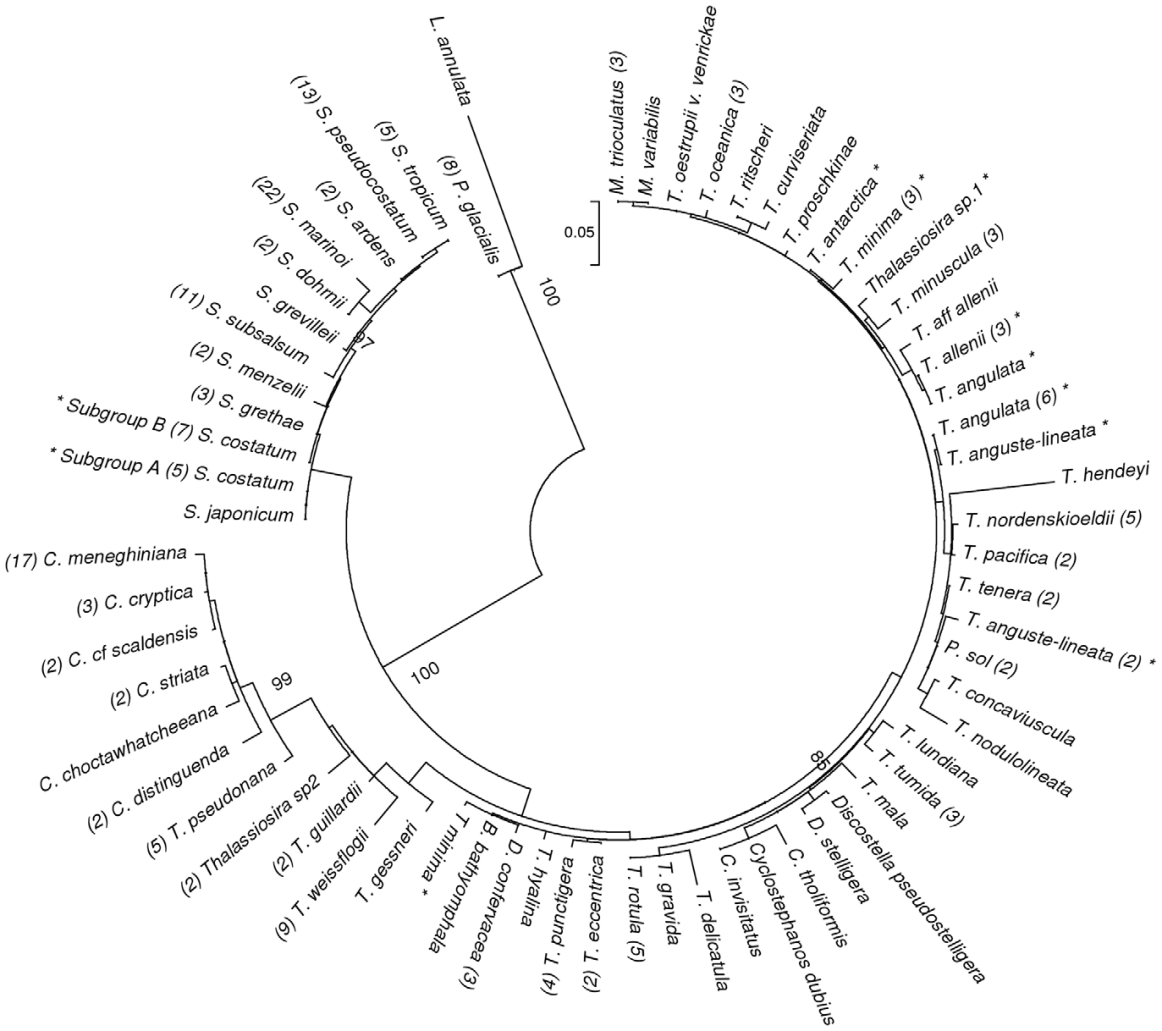
Maximum-likelihood tree for the order Thalassiosirales. The phylogenetic tree was constructed using the Tamura-3-parameter model with all unique V4-region sequences. Boot strap values (1000 replicates) above 80% are shown at branch nodes. Identical sequences were removed from analysis and the representative sequence referenced in Table S1. The number of identical sequences per species is listed in brackets and problem pairs discussed in the text are indicated by an *.

**Figure 2:**
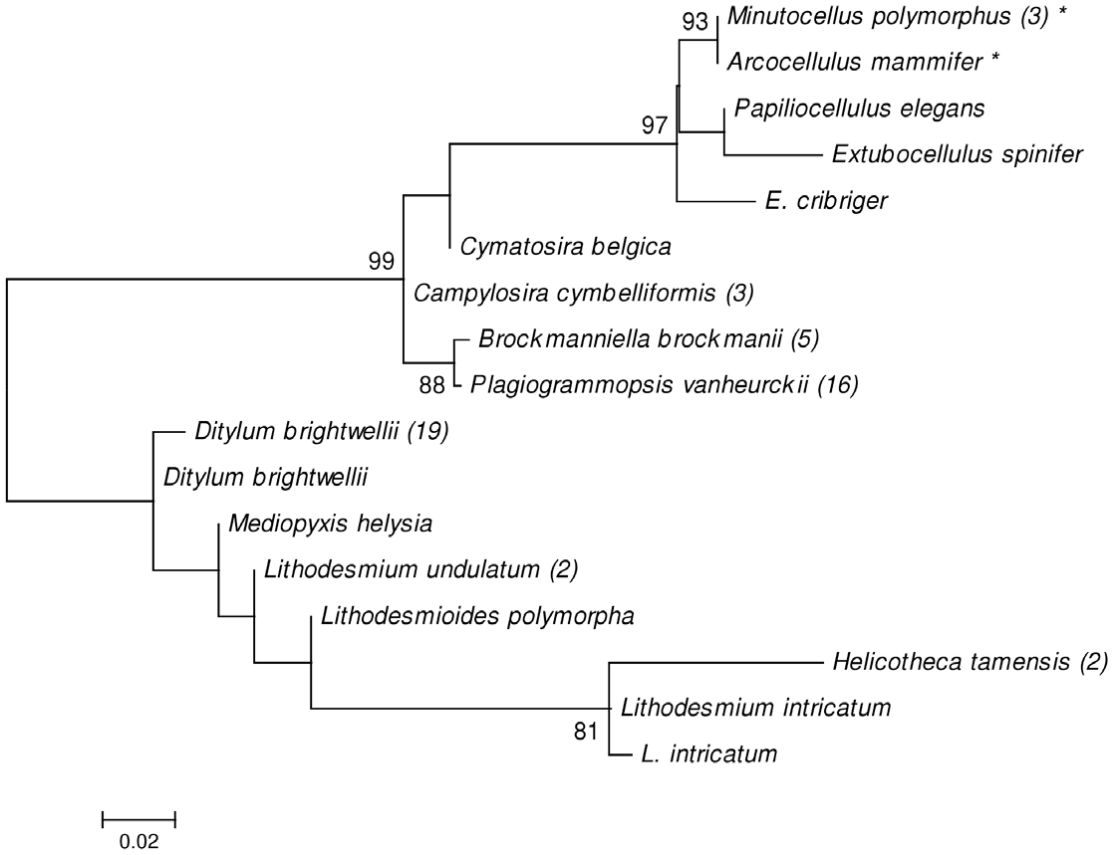
Maximum-likelihood trees for the orders Cymatosirales and Lithodesmiales. The phylogenetic tree was constructed using identical parameters to the Thalassiosirales tree. Bootstrap values (1000 replicates) above 80% are shown at branch nodes. Identical sequences were removed from analysis and the representative sequence referenced in Table S1. The number of identical sequences per species is listed in brackets and problem pairs discussed in the text are indicated by an *.

Intraspecific uncorrected *p-*distances for all species tested ranged from 0 to 0.007 with an average of 0.0014. The highest value of 0.012 was found between the strains of *Cyclotella meneghiniana*. Forty five species which had multiple strains were included in this analysis for a total of 232 sequences. Interspecific uncorrected *p-*distances for all 76 species ranged from 0 to 0.248 with an average *p-*distance of 0.076. Very low (p<0.004) interspecific distances were observed between each of the following pairs (**Fig. 1** **)**: between *Cyclostephanos dubius* and *C*. *invisitatus; Skeletonema grethae* and *S*. *costatum, S. pseudocostatum* and *S*. *tropicum; S*. *ardens* and *S*. *pseudocostatum; S*. *ardens* and *S*. *tropicum; S*. *costatum* and *S*. *tropicum, Minidiscus variabilis* and *M*. *trioculatus; T*. *gravida* and *T*. *rotula; T. tenera* and *T*. *pacifica; T. oestrupii* v. *venrickae* and *M*. *trioculatus* and *M*. *variabilis;* (**Fig. 2**) *Arcocellulus mammifer* and *Minutocellus polymorphus* and *Plagiogrammopsis vanheurckii* and *Brockmanniella brockmannii*. This distance represents 0–2 nucleotide differences over the 333 bp of the fragment tested.

For comparison, the V4 region from sexually compatible clones established from geographically distant locations was sequenced for 10 clones of *Tabularia fasiculata* (Fragilariales) from the West and East Coasts of Canada and from Ukraine, and for 3 clones of *Campylosira cymbelliformis* (Cymatosirales) from the West Coast of the USA, the East Coast of Canada and England, which were also compared with a Gulf of Mexico sequence deposited in Genbank. The average *p-*distance was 0.001 (average of 0.33 nucleotides in the entire V4 region) for *Tabularia fasiculata* (two Pacific clones separate from all others). All *Campylosira cymbelliformis* clone sequences showed 100% identity.

**Figure 3:**
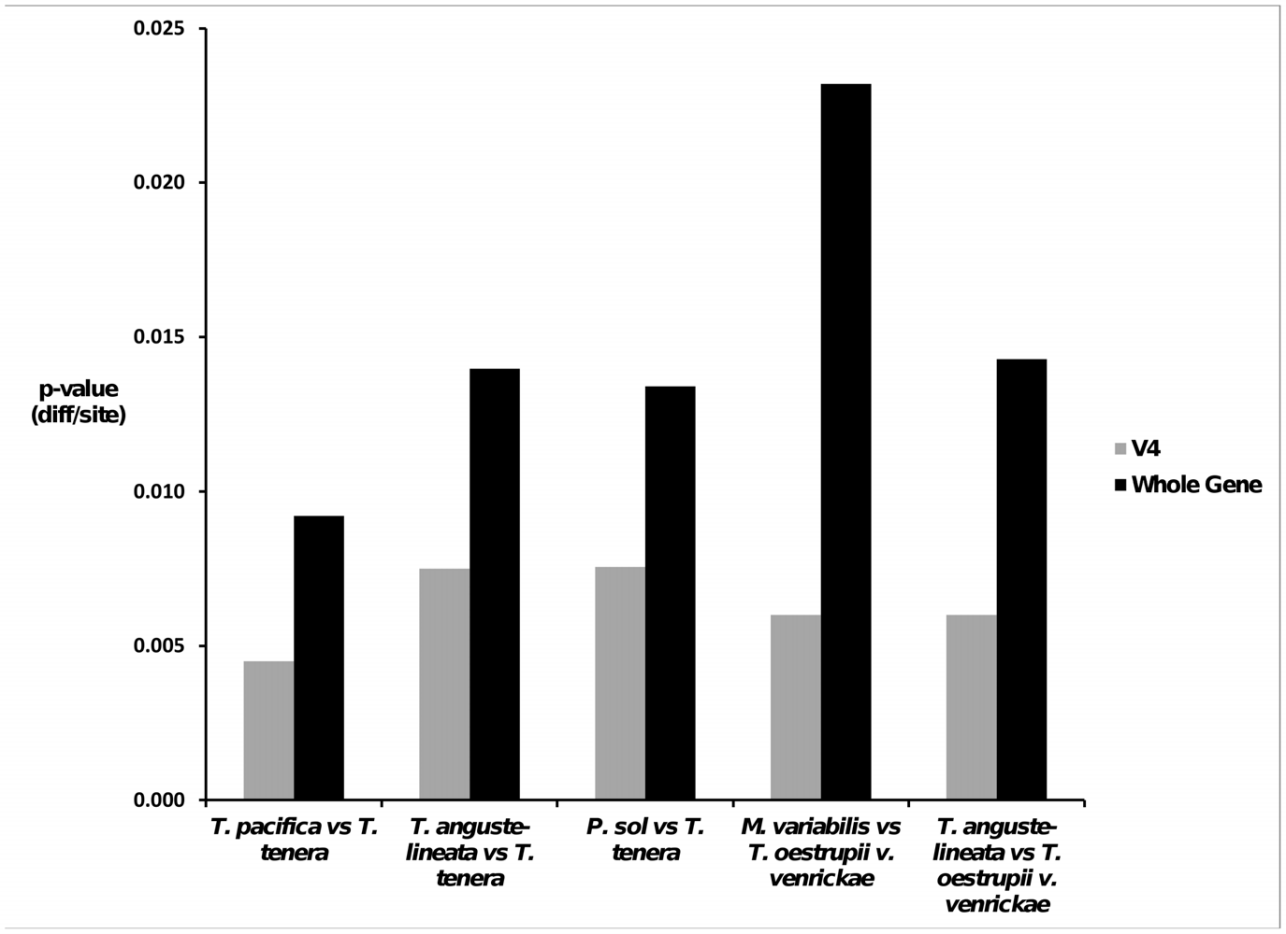
Comparison of the variability of the V4 region barcode used in this study (333 bp) with a 1682 bp fragment representing most of the full 18S gene. All comparisons represented are between one sequence each of the same strain/clone. Differences are represented by the proportion of differences between the sequence pair (*p-*value).

In some species pairs little to no variability was seen in the V4 region but much higher variability was found when much longer (ca. 1680 bp) 18S gene sequences were compared for the same pairs (**Fig. 3**). For instance, *M*. *trioculatus* and *T*. *oestrupii* v. *venrickae* showed a *p-*distance of only 0.003 or 2 nucleotides in the V4 region whereas the full 18S sequences showed a *p-*distance of 0.023 or 39 nucleotides, 6 of which were in the V9 region. Other comparisons that showed this trend, though not as pronounced, were *Planktoniella sol* and *T*. *tenera* which differed from 0.009 in the V4 region to 0.0132 in the full-length 18S sequence, *T*. *angulata* and *T*. *tenera* which differed from 0.006 (V4) to 0.0133 (full 18S) and *T*. *tenera* and *T*. *pacifica* which differed 0.003 (V4) to 0.0087 (full 18s) though in these cases the V9 region did not show higher variability than the V4 as with *M. trioculatus* and *T. oestrupii* v. *venrickae*.

Intergeneric distances were analyzed among the 26 genera based on 264 sequences. The average intergeneric *p-*distance was 0.0873 and ranged from 0.001 to 0.235. The lowest distance (*p = *0.001) was between the two cymatosiroid genera *Arcocellulus* and *Minutocellus*.

The maximum-likelihood trees (**Fig. 1** **&** **2**) constructed from unique sequences of all three orders: Thalassiosirales, Lithodesmiales and Cymatosirales recovered topology generally similar to those proposed earlier [35] and showed resolution of most species. The species not resolved were those whose *p-*values were low as reported above.

### Reassessment of Sequence Identities for Distance Analysis

An instructive preliminary inspection of the Thalassiosirales tree **(** **Fig. 1**) revealed that several sequences with the same name retrieved from GenBank were clearly separated from others, while in other cases 100% identical (in V-4 region) sequences carried different names. Prior to distance analysis we attempted to resolve some of these anomalies by re-examination of either sequences or images associated with the sequences. The conclusions of this examination are summarized in **Table 1**.

**Table 1:** Summary of the reassessment of sequence identities.

Accession Number	Strain Code	Published or GenBank Name	Morphological Species
JX437374	CCMP982	*Thalassiosira antarctica*	*Thalassiosira antarctica*
EF140621	T1	*Thalassiosira antarctica*	*Thalassiosira* sp.1
DQ093366	CCMP991	*Thalassiosira minima*	*Bacterosira* sp.
DQ514876	CCMP990	*Thalassiosira minima*	*Thalassiosira minima*
DQ514867	BEN02-35	*Thalassiosira angulata*	*Thalassiosira allenii*
AJ810854	MHta1	*Thalassiosira anguste-lineata*	*Thalassiosira angulata*
JX437386	IIIB3	*Thalassiosira angulata*	*Thalassiosira angulata*
HM991688	DDZ-2010a	*Thalassiosira allenii*	*Thalassiosira allenii*

The names used here correspond to the morphological identity from new or published SEM images (see text for details).

**Figure 4:**
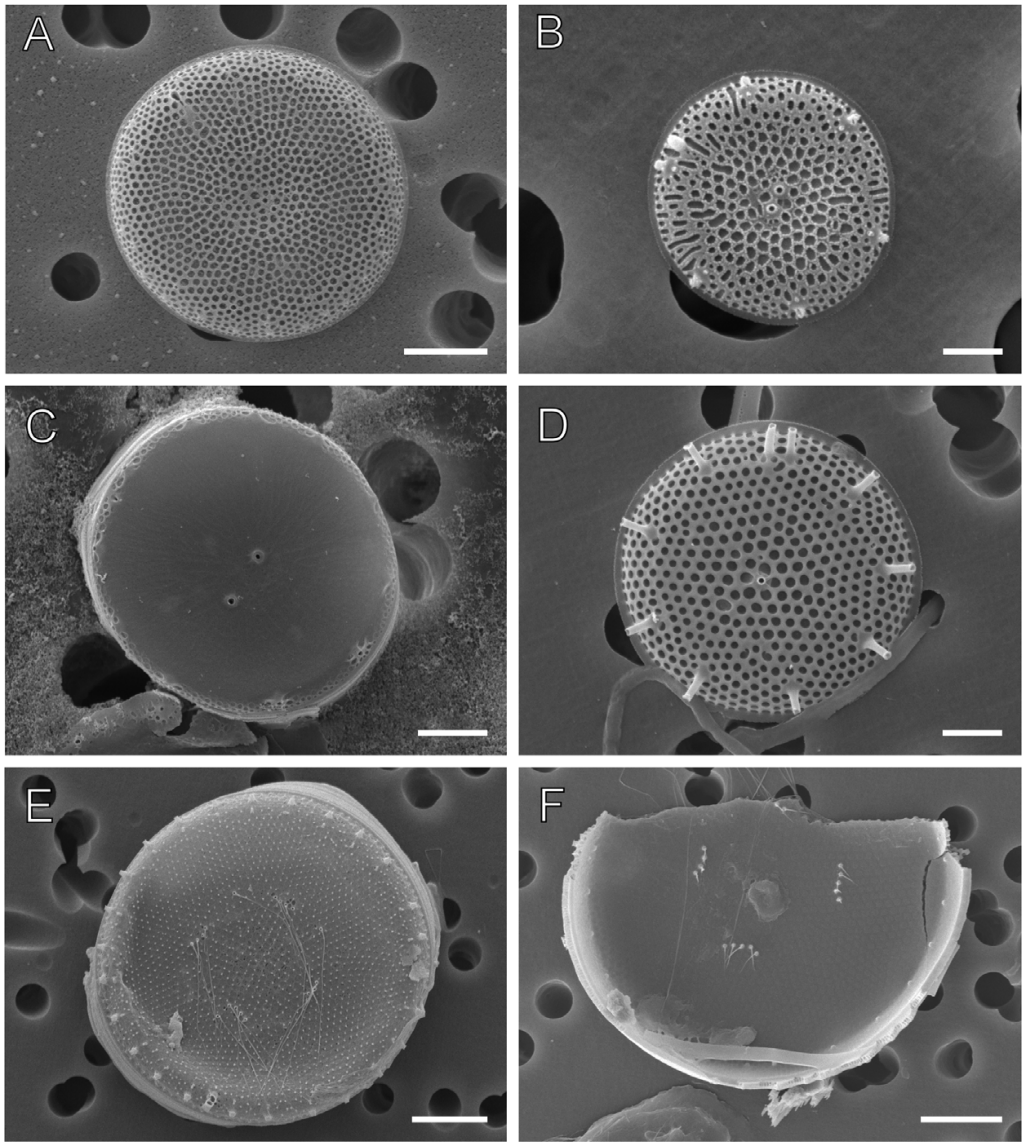
SEM images of *Thalassiosira* species used in this study for the reassessment of sequence identity for ambiguous sequences. Scale bars = 3 µm (A), 1 µm (B), 2 µm (C,D) and 5 µm (E,F). (**A**) *Thalassiosira antarctica* strain CCMP 982 (**B**) *Thalassiosira minima* strain CCMP 985 (**C**) *Thalassiosira “minima”* strain CCMP 991 (**D**) *Thalassiosira angulata* clone PCB2 (E-F) *Thalassiosira anguste-lineata* isolate Art-22/127 from post-PCR SEM, not acid cleaned.

Firstly, two sequences identified as *Thalassiosira antarctica* (CCMP982:JX437374 and T1:EF140621) grouped into separate clades and were distinct in terms of genetic distance with *p = *0.012 diff/site, thus far exceeding most of the intraspecific distances in our data set. One sequence (CCMP982) clustered with *T. minima* whereas the other (currently named *Thalassiosira* sp 1, T1) grouped with *Thalassiosira minuscula* (**Fig. 1**). CCMP982 had been cultured in our Mount Allison laboratory some years ago and SEM images (**Fig. 4A** **)** available showed that this clone morphologically conformed to T. *antarctica* (metric data: mean±SEM of the following characters: diameter, d = 14.33±0.16, valve face areolae in 10 µm a = 23.81±0.57 and fultoportulae in 10 µm f = 7.38±0.32, metrics will follow this order below) as per Hasle and Heimdal [37] albeit cells in our culture were at or below the species range for valve diameter and with no evidence of spores. Therefore, we conclude that CCMP982 is likely *T. antarctica* while the strain T1 represents a species for which an identified reference sequence is not yet available.

Secondly, two sequences attributed to *T. minima* (DQ093366.1, CCMP991 and DQ514876, CCMP990) differed phylogenetically as well. The former was closest to *Bacterosira bathyomphala* while the latter was closest to CCMP985 (JX437382), *Thalassiosira floridana* (currently synonymized to *T. minima* in Hasle [38]. Again, two of these strains had been grown earlier in our Mount Allison lab and we have SEM images for CCMP985 (**Fig. 4B** **)** and CCMP991 (**Fig. 4C** **)**. They both fit the morphological descriptions of *T. minima* by Hasle [38] with two central strutted processes and in terms of measurements for diameter (5.97±0.12 µm for CCMP985 and 10.2±0.6 µm for CCMP991), areolae in 10 µm (37.29±1.23 for CCMP985 and 42.1±8.4 for CCMP991) and number of fultoportulae in 10 µm (4.48±0.07 for CCMP985 and 4.5±0.8 for CCMP991) though cultured CCMP991 shows no distinct areolae throughout the valve face except at the margin. Because of the sequence differences and the lack of areolation on the valve face in CCMP991 we concur that this strain may represent a novel species related to the genus *Bacterosira* as suggested by Alverson *et al.* [39].

Even more convoluted seems the case involving four other sequences (DQ514867, AJ810854, JX437386 and HM991688) summarized in **Table 1**. These are identified as “*Thalassiosira angulata”* [39], “*T. anguste-lineata”* [40], T. *angulata* [21], [41] and *T. allenii* (GenBank direct submission). The first of these sequences, “*T. angulata”* (DQ514867) is identical to the fourth, *T. allenii* (HM991688), and is different from our clones of *T. angulata* (**Fig. 4D**) which have metrics (as ordered above) of d = 12.13±0.26 µm, a = 18.43±0.43/10 µm and f = 2.79±0.03/10 µm consistent with the type [42]. SEM images of this clone BEN02-35 (DQ514867) retrieved from ProtistCentral also differ from the specimens of *T. angulata* by the areolae on the valve mantle being smaller then on the valve face, a character attributed to *T. allenii* [42]. Consequently, we assigned this clone to *T. allenii* for this study.

The second sequence (AJ810854) deposited as “*T. anguste-lineata”* is, however, identical to our clones of *T. angulata* from the Canadian Maritimes [21] represented by sequence 3 (JX437386). This is an intriguing case because we have 5 clones of *T. angulata* sequenced and morphologically they all meet the specific diagnostic morpho-criteria of Hasle [42] attributed to this species. However, the SEM images presented in Hoppenrath *et al.* [40] seem to represent *T. anguste-lineata* [43] though with clusters of only one to two central fultoportulae. Furthermore, the sequence and images retrieved from our own isolate chains of *T. anguste-lineata* Art-22 and Art-137 (**Fig. 4E–F** **)** also conform well to the type description [43] (as per measurements of d = 28.16±0.41 µm, a = 14.17±1.17/10 µm and f = 3.22±0.11/10 µm) and morphological and sequence data from Alverson *et al.* [39] and differ considerably from AJ810854. We therefore conclude that this sequence represents *T. angulata,* a conclusion shared by the authors (Hoppenrath M & Beszteri B, pers. comm.). These conclusions (Table S1) were applied to distance metrics and are incorporated in **Fig. 1**.

### Character Analysis

**Figure 5:**
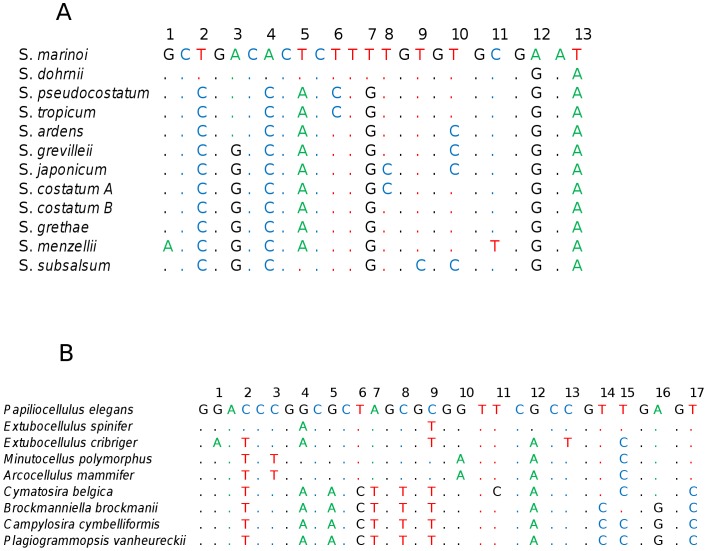
Variable sites used as diagnostic characters in the V4 region of select sequences. (**A**) sequences from the genus Skeletonema and (**B**) sequences from the order Cymatosirales. Numbers above sequences represent variable sites and points represent sequence identity with the top sequence. Conserved regions between variable sites were collapsed for brevity.

A character based analysis was performed on the 11 species from the genus *Skeletonema* as well as from the order Cymatosirales (**Fig. 5**). These groups were chosen because of their very low intraspecific genetic distances. For members of the genus *Skeletonema* all species but 2 were separated by this approach. Three species (*S. menzelli, S. grevillei,* and *S. marinoi*) were readily separated using single, pure diagnostic characters (transition or transversion unique to a species). *Skeletonema pseudocostatum* and *S. tropicum,* two species which showed no genetic distance between them were separated as a pair from all others by a single diagnostic character as well. In addition, four other species were separated by compound pure diagnostic characters, *S*. *japonicum, S. subsalsum, S. ardens*, and *S. dohrnii*. Further, *S. costatum* and *S. grethae* were separated as a pair from all others by a double compound pure diagnostic character. Finally, *S*. *costatum* sequences were separated into two subgroups: those identical to *S. grethae* (**Fig. 1** **Subgroup B**) and those separated by a compound diagnostic character from *S. grethae* (**Fig. 1** **Subgroup A**). In total nine of the *Skeletonema* species were separated using a character based approach while two species, *S. grethae* and *S. tropicum* remained inseparable. Many private characters (unique to one or more sequences but not the entire group/species) were also found in different GenBank sequences of various *Skeletonema* species exhibiting no particular pattern with respect to type of change (i.e. transition or transversion) or geography.

This approach was equally successful for the members of the order Cymatosirales showing extremely low divergence in the V4 region. Four species were separated by single, pure diagnostic characters (*Cymatosira belgica, Extubocellulus cribriger, E. spinifer* and *Minutocellulus polymorphus*) and four more species were separated by compound pure diagnostic characters, (*Plagiogrammopsis vanheurckii*, *Brockmanniella brockmannii*, *Campylosira cymbelliformis* and *Papiliocellulus elegans*). In this case the only species out of nine included in our dataset that did not have a pure diagnostic character in the tested fragment was *Arcocellulus mammifer* which was identical to *M. polymorphus* in the V4 region.

### Barcode Threshold

All sequences in this study were examined for how well they separated species according to a threshold value. A threshold of p = 0.02 successfully separated 96.9% of species tested. The efficacy of the marker increased when the threshold level was reduced to p = 0.01 (3 nucleotides) with 99.6% of all species separated. However, this lower threshold resulted in the overlap of six intraspecific values (of 45 species groups) and thus indicates that a threshold lower than p = 0.02 would be too low to retain necessary taxonomic information at the species level.

## Discussion

### Amplification and Sequencing

A useful DNA barcode should amplify reliably, be universal for all taxa at the taxonomic level chosen and be variable enough to separate species. The V4 region of the 18S gene amplified and sequenced successfully for all cultured strains and for nearly half of single chains. This ease of amplification and sequencing across the taxa was also reported by Zimmermann *et al.* [22] and indicates that the V4 region primers are sufficiently universal and the V4 locus is readily accessible. Thus, this DNA fragment met the first two criteria of an effective barcode. The poorer success rate for our single chains was most likely a consequence of the small amount of genetic material available from the few cells in the chain seen also for other multiplexed targets [28].

Importantly for the application of a candidate barcode, Zimmermann *et al.* [22] noted that the 18S gene already has a strong representation in GenBank. This history of record provides the needed reference sequence base with which to compare unknown, newly retrieved sequences that otherwise could not be identified. Unlike the entire 18S sequence or other barcodes, however the V4 region is short and thus can be easily retrieved from small or precious samples as well as from fixed or heavily degraded DNA similar to cox1 “minibarcodes” [44]. Recent high throughput amplicon sequencing efforts have also focused on the V4 region [45] and increased taxonomic resolution of these sequences would be a significant benefit.

The V4 region amplified included flanking homologous base pairs that were readily aligned. This contrasts the 5.8S, ITS2 barcode region [21] where specific parameters and conserved anchor points are needed to produce a confident alignment. This ease of alignment means that little editing is required to accurately represent sequence homology. Here, even the three sequences with large inserts (*Extubocellulus cribriger*, *E. spinifer* and *Cymatosira belgica*) were readily aligned with all other sequences as the inserts occurred downstream of the V4 region and flanking conserved regions and were removed.

### Distance Analysis

Intraspecific uncorrected *p-*distances expressed as the proportion of differing sites between two sequences were on average much lower than interspecific distances which represent a characteristic of a good DNA barcode. Some species pairs showed very low interspecific *p*-distances (problem pairs listed above) and some of them were also morphologically semi-cryptic. Zimmermann *et* *al.* [22] already found that some members of the genus *Stephanodiscus* showed little divergence and the same pattern was seen for sister clades in our study. For example, *Cyclostephanos dubius* and *C. invisiatus* showed no difference in the V4 region. Among our new sequences, those from the genus *Skeletonema* were particularly troublesome in terms of species separation by the V4 region. Among these the *p-*distances with very little resolution were the following species pairs: *Skeletonema grethae* and *S. costatum* (0.003), *S. pseudocostatum* and *S. tropicum* (0.001), *S. ardens* and *S. pseudocostatum* (0.007) and *S. ardens,* and *S. tropicum* (0.006).

Until recently, however, the genus *Skeletonema* included only a few species: *S. costatum, S. tropicum*, *S. subsalsum*, *S. potamos*, *S. cylindraceum* and *S. menzelii,* though in most cases field samples containing *Skeletonema* are identified as “*S. costatum”* [4]. This attests to the semi-cryptic nature of these species’ morphology. Sarno *et al.* [4] applied extensive morphological analysis in conjunction with a molecular assessment of the 18S and 28S rDNA genes and found evidence supporting the segregation of the *Skeletonema costatum*-complex into four new species (*S. dohrnii*, *S. grethae*, *S. japonicum* and *S. marinoi*). Sarno *et al.* [46] though, also found that the entire 18S gene poorly resolved the relationships between some of the *Skeletonema* species and thus the V4 region alone may also be insufficient in resolving all morpho-species in this complex without a companion marker.

*Mindiscus variabilis* and *M. trioculatus* were also identical over the V4 region, however, some genetic distances between these two morpho-species were previously reported using the whole 18S gene as well as the ITS region [47], the two species were also relatively easy to separate morphologically and the separation of the two species was justified on that basis.

Among the 32 *Thalassiosira* species there were three problem pairs: *Thalassiosira gravida* and *T.* *rotula*; *T.* *tenera* and *T.* *pacifica*; *T.* *oestrupii v.* *venrickae* and *M.* *trioculatus* as well as *M.* *variabilis*. The difference between *T.* *gravida* and *T.* *rotula* was very small (p = 0.00362). This may reflect the long standing debate as to whether the two are distinct species or the same species with varying morphologies [48], [49], and may support the latter. The other species pairs representing a more interesting case and will be discussed further below.

Within the order Cymatosirales *Arcocellulus mammifer* and *Minutocellus polymorphus* were identical and *Plagiogrammopsis vanheurckii* and *Brockmanniella brockmannii* differed by only p = 0.003 over the region tested, suggesting the V4 region could be a poor barcode for genera in this order based on distance methods alone though in this order the dataset was limited.

Biologically defined species used in this study, those with separate mating types from the genera *Tabularia* and *Campylosira* were represented by clones isolated from sites across continents and oceans and showed very low genetic distances. These very low genetic distances may be expected among representatives of panmictic (random mating) populations. One sequence of *C. cymbelliformis* from GenBank showed 100% sequence similarity to the other clones suggesting perhaps genetic conservatism in this locus independent of geography or that there is ongoing genetic exchange via long range transport between North Pacific, North Atlantic and the Caribbean.

Some species pairs in our test-set exhibited very low variability in the V4 region but much higher variability over the entire 18S sequence (e.g. *Minidiscus trioculatus* and *T. oestrupii* v. *venrickae*, *Planktoniella sol* and *T. tenera, T. angulata* and *T. tenera* and *T. tenera* and *T. pacifica*). While our study only examined diatoms, a recent study by Dunthorn *et al.* [23] compared the V4 region with the alternate hyper-variable region V9 in ciliates and came to the conclusion that the V4 region also more closely approximates the variability of the entire gene in agreement with Zimmermann *et al.* [22]. While this suggests the phenomenon is not widespread, our observation nonetheless illustrates a possible shortcoming for a V4 based barcode and emphasizes the need for further taxon sampling among diatoms to establish whether the phenomenon is common.

### Character Analysis

Applying a character based approach to DNA barcoding at the level of genus resolved some of the ambiguities in the distance method. In the case of *Skeletonema* all species with the exception of S. *tropicum* and S. *grethae* were separated. The private characteristics observed in various GenBank *Skeletonema* sequences that did not correspond to species or subgroups were consistent with sequence base-calling error. Additionally, the application of character analysis may have clarified sequence relationships between subgroups of *S*. *costatum.* Two groups of S. *costatum* sequences, subgroup A and B were recoverd. Subgroup A was distinguised from subgroup B containing *S. grethae* and several GenBank sequences attributed to *S*. *costatum* (CCMP1077/4 A-D, 2A–2D) by a pure diagnostic character. This suggests that *S*. *costatum* subgroup B and S. *grethae* may be conspecific. This is consistent with the fact that one S. *grethae* sequence (X85395) in this subgroup belongs to a specimen which was first attributed to *S*. *costatum* based on morphology [50] but then subsequently transferred to *S*. *grethae* [4]. Similarly, it may be that the other sequences attributed to S. *costatum* in Alverson & Kolnick [51] of subgroup B are in fact *S*. *grethae*. If so, the two species can be separated by character analysis.

As with the genus *Skeletonema,* character analysis in Cymatosirales resolved several of the ambiguities resulting from genetic distance analysis alone. All species in the order showed pure diagnostic characters (single and compound) that distinguished them, including *Plagiogrammopsis* and *Brockmanniella brockmannii* whose separation was justified by a CC-CT difference in the 14^th^ and 15^th^ variable sites. The only exception was *Arcocellulus mammifer* whose separation from *Minutocellus polymorphus* could not be justified based on our marker.

### Barcode Threshold

A key test for a DNA barcode is its efficacy in separating species according to a threshold value. In this study, a threshold of p = 0.02 was found to separate 96.9% of all species. Zimmermann *et al.* [22] found that all but the members of *Stephanodiscus* were successfully separated using V4 as a barcode. Though Zimmermann *et al.* [22] did not define a species threshold value intrageneric distances of greater than 0.029 suggest that a threshold of 0.02 sufficiently separated the majority of species except for the genera *Mayamaea* (0.01) and *Stephanodiscus* (0.001). In comparison to other proposed barcode markers, the V4 region fares well. The rbcL barcode tested resolved only 90% of species tested at a threshold of *p* = 0.02 [20] and the 5.8S-ITS2 barcode marker proposed separated 95% of all species tested using a much higher threshold of *p* = 0.11 and with a much larger dataset [21]. Therefore, the V4 region performed better than rbcL and similarly to 5.8S-ITS2. The much higher threshold value seen in the 5.8S-ITS2 barcode [20] suggests that it could be used as a conxurrently amplified companion marker for situations where V4 alone does not resolve species.

Aside from the few problem pairs discussed above the majority of thalassiosiroid morpho-species were successfully separated by a V4 barcode through distance methods alone. Additionally, through the application of a character based analysis a better separation (97.5% vs. 96.9%) of species was achieved and a better still separation would likely result from the application of this method to the order Thalassiosirales which would likely require more sequences per species and an additional step utilizing existing software [52] to handle the high number of similar sequences.

### Conclusions

Our findings support those of Zimmermann *et al.* [22] that the V4 region could serve as an effective barcode for diatoms as it separated 96.9% of all tested species. Some species for which there was little separation in the V4 region were then separated by additional character analysis. We suggest that due to the ease of amplification, the extensive database of 18S sequences and resolving power, the V4 region of the 18S gene would be a suitable barcode marker for diatoms. Furthermore, we advocate a combined approach using distance methods, tree building and character based analysis for species identification of diatoms using DNA barcodes especially for cases of closely related or otherwise difficult to segregate species.

## Supporting Information

Table S1 **Strains and clones used in this study, their representative geography accession numbers and BOLD accession numbers for sequences generated in this study when available.** Strains in bold are those included in the phylogenetic trees representing multiple identical sequences; accession numbers in bold indicate sequences generated in this study and strains whose taxonomic affinity is discussed in the text are indicated by †.
(DOCX)
