# Supplementary material for: Distance and Character-Based Evaluation of the V4 Region of the 18S rRNA Gene for the Identification of Diatoms (Bacillariophyceae)
Source: PLoS One. 2012 Sep 21;7(9):e45664. doi: 10.1371/journal.pone.0045664 (PMC3448646; doi:10.1371/journal.pone.0045664)
Supplement: Table S1 — Strains and clones used in this study, their representative geography accession numbers and BOLD accession numbers for sequences generated in this study when available. Strains in bold are those included in the phylogenetic trees representing multiple identical sequences; accession numbers in bold indicate sequences generated in this study and strains whose taxonomic affinity is discussed in the text are indicated by †. (DOCX) [file pone.0045664.s001.docx]

Table S1. Strains and clones used in this study, their representative geography accession numbers and BOLD accession numbers for sequences generated in this study when available. Strains in bold are those included in the phylogenetic trees representing multiple identical sequences; accession numbers in bold indicate sequences generated in this study and strains whose taxonomic affinity is discussed in the text are indicated by †.

| Species | Strain | Geography | Accession Number | BOLD Accession Number |
| --- | --- | --- | --- | --- |
| **Lithodesmiales** | | | |  |
| *Ditylum brightwelli* (West) Grunow | CCAP1022/2 | Unknown | X85386.2 |  |
| *D. brightwellii* | 278 | Puget Sound, USA | AY188181.1 |  |
| *D. brightwellii* | ADM2M2 | Puget Sound, USA | DQ329269.1 |  |
| *D. brightwellii* | CCMP358 | Gulf of Mexico | AY485444.1 |  |
| *D. brightwellii* | GG21 | Puget Sound, USA | FJ266029.1 |  |
| *D. brightwellii* | GG22 | Puget Sound, USA | FJ266030.1 |  |
| *D. brightwellii* | WBD3 | Puget Sound, USA | FJ266033.1 |  |
| *D. brightwellii* | EAP1J | Puget Sound, USA | DQ329271.1 |  |
| *D. brightwellii* | EAP1Y50 | Puget Sound, USA | DQ329270.1 |  |
| *D. brightwellii* | P2A4 | Puget Sound, USA | AY188182.1 |  |
| *D. brightwellii* | PDB3 | Puget Sound, USA | FJ266031.1 |  |
| *D. brightwellii* | PDB5 | Puget Sound, USA | FJ266032.1 |  |
| *D. brightwellii* | PSB3M 1 | Puget Sound, USA | DQ329268.1 |  |
| *D. brightwellii* | SMDC01 | Incheon coastal, South Korea | EU364891.1 |  |
| *D. brightwellii* | SMDC02 | Incheon coastal, South Korea | EU364892.1 |  |
| *D. brightwellii* | WBD4 | Puget Sound, USA | FJ266034.1 |  |
| *D. brightwellii* | CCMP1582 | Renesee, The Netherlands | **JX437437** | DITS151-08 |
| *D. brightwellii* | SFPB44A1 | Passamaquody Bay, Canada | **JX437438** | DITS094-08 |
| *D. brightwellii* | **CCMP359** | George’s Bank, Atlantic | **JX437439** | DITS222-08 |
| *D. brightwellii* | **CCMP361** | La Jolla, USA | **JX437440** | DITS220-08 |
| *Helicotheca tamensis* (Shrubsole) M. Ricard | **CCMP1687** | Gulf of Oman, Arabian Sea | **JX437435** | DITS201-08 |
| *H. tamensis* | CCMP825 | Unknown | **JX437436** | DITS209-08 |
| *Lithodesmioides polymorpha* von Stosch | **ECT3772** | Taelayag Beach, Guam | HQ912655.1 |  |
| *Lithodesmium intricatum* (T. West) H. & M. Peragallo | **ECT3836** | Long Beach, USA | HQ912670.1 |  |
| *L. intricatum* | **ECT3850** | Long Beach, USA | HQ912678.1 |  |
| *L. undulatum* Ehrenberg | CCMP1797 | Daytona Beach, USA | HQ912559.1 |  |
| *L. undulatum* | **CCMP1806** | Unknown | DQ514846.1 |  |
| *Mediopyxis helysia* Kühn, Hargreaves & Halliger | **N/A** | Helgoland, Germany | AJ968728.1 |  |
| **Thalassiosirales** | | | |  |
| *Bacterosira bathyomphala* (Cleve) E.E. Syverten & G.R. Hasle | **NB04-B6** | Narragansett Bay, USA | DQ514894.1 |  |
| *Cyclotella cf scaldensis* Muylaert & Sabbe | **G18W53** | Geeste estuary, Germany | AY496208.1 |  |
| *C. cf scaldensis* | G1W11 | Geeste estuary, Germany | AY496209.1 |  |
| *C. choctawhatcheeana* Prasad | **L1840** | Geeste estuary, Germany | AM712618.1 |  |
| *C. cryptica* Reimann, Lewin & Guillard | CCMP331 | Unknown | **JX437396** | DITS184-08 |
| *C. cryptica* | **CCMP332** | Martha's Vineyard, USA | **JX437397** | DITS183-08 |
| *C. cryptica* | CCAP1070/2 | Unknown | AY485499.1 |  |
| *C. distinguenda* Hustedt | **N/A** | Tiplady Bog, USA | DQ514859.1 |  |
| *C. menegheniana* Kützing | F8 | Fugleso Lake, Denmark | DQ514853.1 |  |
| *C. menegheniana* | L1263 | Stump Lake, USA | DQ514854.1 |  |
| *C. menegheniana* | TI1 | Lake Titicaca, Peru | DQ514860.1 |  |
| *C. meneghiniana* | p567 | Black Sea | AJ535172.1 |  |
| *C. meneghiniana* | G17W8 | Geeste estuary, Germany | AY496213.1 |  |
| *C. meneghiniana* | HYK0210-A5 | Han River, South Korea | GQ148716.1 |  |
| *C. meneghiniana* | AT-cm13 | Unknown | AM236073.1 |  |
| *C. meneghiniana* | SAG 1020-1a | River Werra, Germany | AY221723.1 |  |
| *C. meneghiniana* | G18W41 | Geeste estuary, Germany | AY496206.1 |  |
| *C. meneghiniana* | G188D | Geeste estuary, Germany | AY496207.1 |  |
| *C. meneghiniana* | G17W3 | Geeste estuary, Germany | AY496210.1 |  |
| *C. meneghiniana* | G8W7 | Geeste estuary, Germany | AY496211.1 |  |
| *C. meneghiniana* | G8W4 | Geeste estuary, Germany | AY496212.1 |  |
| *C. meneghiniana* | **CCMP 337** | Bét She'an Valley, Israel | DQ093371.1 |  |
| *C. meneghiniana* | HYK0210-A1 | Han River, South Korea | GQ148712.1 |  |
| *C. meneghiniana* | HYK0210-A3 | Han River, South Korea | GQ148714.1 |  |
| *C. meneghiniana* | Waco1 | Lake Waco, USA | HQ912576.1 |  |
| *C. striata* Kützing (Grunow) | **CCMP1586** | Jakarta Harbour, Indonesia | DQ514851.1 |  |
| *C. cf striata* | CCMP1577 | Jakarta Harbor, Indonesia | **JX437398** |  |
| *Discostella pseudostelligera* (Hustedt) Houk & Klee | **ROR01-1** | Raccourci Old River, USA | DQ514905.1 |  |
| *D. stelligera* (Cleve & Grunow) Houk & Klee | **L1360** | Big Pond, Cedar Hill, USA | DQ514903.1 |  |
| *Cyclostephanos dubius* (Hustedt) Round | **Waco5** | Lake Waco, USA | HQ912575.1 |  |
| *C. invisitatus* (Hohn & Hellermann) Theriot, Stoermer & Håkasson | **FHTC26** | Fairport Harbor, USA | DQ514899.1 |  |
| *C. tholiformis* Stoermer, Håkansson & Theriot | **FHTC15** | Fairport Harbor, USA | DQ514898.1 |  |
| *Porosira glacialis* (Grunow) Jørgensen | CCMP1099 | Zavodovski Island, Antarctica | **JX437399** | DITS123-08 |
| *P. glacialis* | CCMP1274 | Weddell Sea, Antarctica | **JX437400** | DITS118-08 |
| *P. glacialis* | CCMP670 | Zavodovski Island, Antarctica | **JX437401** | DITS137-08 |
| *P. glacialis* | CCMP980 | South Georgia Island | **JX437402** | DITS132-08 |
| *P. glacialis* | CCMP315 | Grise Fjord, Canada | EF585583.1 |  |
| *P. glacialis* | CCMP668 | Atlantic, New Hampshire, USA | HQ912619.1 |  |
| *P. glacialis* | **Art29** | South Shore, Atlantic, Canada | **JX437403** |  |
| *P. glacialis* | N/A | Unknown | EF192992.1 |  |
| *Planktoniella sol* (C.G.Wallich) Schütt | **CCMP1608** | Mid Atlantic | HQ912562.1 |  |
| *P. sol* | p354 | Indian Ocean | AJ535173.1 |  |
| *Lauderia annulata* (Cleve) | **CS30** | La Jolla, USA | DQ514849.1 |  |
| *Detonula confervacea* (Cleve) Gran | CCMP353 | Narragansett Bay, USA | HQ912617.1 |  |
| *D. confervacea* | N/A | Unknown | EF192991.1 |  |
| *D. confervacea* | **CCMP352** | Dennis, USA | **JX437371** | DITS141-08 |
| *Minidiscus trioculatus* (F.J.R. Taylor) Hasle | **CCMP496** | Unknown | **JX437377** | DITS171-08 |
| *M. trioculatus v. monoculatus* Kaczmarska | MiniNova2 | Passamaquody Bay, Canada | **JX437378** | DITS090-08 |
| *M. trioculatus v. monoculatus* | MiniNova | Bay of Fundy, Canada | FJ590769.1 |  |
| *M. variabilis* Kaczmarska | **CCMP 495** | Gulf of Maine, USA | FJ590771.1 |  |
| *Thalassiosira allenii* Takano | **N/A** | Unknown | HM991688.1 |  |
| *T.* *allenii* (as *T.* *angulata* in Alverson, 2007) † | **BEN02-35** | San Joaquin River, USA | DQ514867.1 |  |
| *T. allenii* | DDZ-2010a | Unknown | HM106505.1 |  |
| *T. aff allenii* | **CCMP986** | Tromso, Norway | **JX437373** | DITS129-08 |
| *T. angulata* (W. Gregory) Hasle | IIIB2 | Bay of Fundy, Canada | **JX437384** | DITS086-08 |
| *T. angulata* | IIC3 | Bay of Fundy, Canada | **JX437385** | DITS084-08 |
| *T. angulata* | **IIIB3** | Bay of Fundy, Canada | **JX437386** | DITS085-08 |
| *T. angulata* | PBC2 | Passamaquody Bay, Canada | **JX437387** | DITS358-08 |
| *T. angulata* | PBC3 | Passamaquody Bay, Canada | **JX437388** | DITS359-08 |
| *T.* *angulata (*as *T*. *anguste-lineata* in Hoppenrath et al. 2007) † | **MHta1** | North Sea | AJ810854.1 |  |
| *T. anguste-lineata* (A. Schmidt) G. Fryxell & Hasle | BEN02-30 | San Joaquin River, USA | DQ514865.1 |  |
| *T. anguste-lineata* | **Art-22** | South Shore, Atlantic, Canada | **JX437394** | VFOUR001-12 |
| *T. antarctica* Comber | **CCMP982** | Oslo Fjord, Norway | **JX437374** | DITS130-08 |
| *T. concaviuscula* Makarova | **MHtc1** | North Sea | AJ810857.1 |  |
| *T. curviseriata* Takano | **MHtcu1** | North Sea | AJ810859.1 |  |
| *T. delicatula* Ostenfeld | **MHtd1** | North Sea | AJ810855.1 |  |
| *T. eccentric* (Ehrenberg) Cleve | **BER02-09** | San Francisco Bay, USA | DQ514868.1 |  |
| *T. eccentrica* | N/A | Unknown | DQ514868.1 |  |
| *T. gessneri* (Hustedt) | **AN02-08** | San Joaquin River, USA | DQ514864.1 |  |
| *T. gravida* Cleve | **CCMP987** | Tromso, Norway | **JX437381** |  |
| *T. guillardii* Hasle | CC03-04 | Clam Creek, USA | DQ514869.1 |  |
| *T. guillardii* | **CCMP988** | Rhode Island, Atlantic, USA | DQ514875.1 |  |
| *T. hendeyi* Hasle & G.Fryxell | **MHth1T** | North Sea | AM050629.1 |  |
| *T. hyalina* (Grunow) Gran | **Art 58** | South Shore, Atlantic, Canada | **JX437383** |  |
| *T. lundiana* Fryxell | **N/A** | Unknown | HM991692.1 |  |
| *T. minima* Gaarder | CCMP990 | Unknown | DQ514876.1 |  |
| *T. minima†* | **CCMP991** | Gulf of Maine | DQ093366.1 |  |
| *T. minima* | **CCMP985** | Pompano Beach, Canada | **JX437382** | DITS208-08 |
| *T. minima* | 7918 | Gulf of Mexico | JF791059.1 |  |
| *T. minuscula* Krasske | **CCMP1093** | La Jolla, USA | DQ514882.1 |  |
| *T. minuscula* | FB02-31 | San Francisco Bay, USA | DQ514887.1 |  |
| *T. minuscula* | N/A | Unknown | HM991694.1 |  |
| *T. nodulolineata* (Hendey) Hasle & Fryxell | **BEN02-33** | San Joaquin River, USA | DQ514866.1 |  |
| *T. nordenskioeldii* Cleve | **1C4** | Shediac Valley, Canada | **JX437390** | DITS362-08 |
| *T. nordenskioeldii* | CCMP997 | Tromso, Norway | **JX437391** | DITS159-08 |
| *T. nordenskioeldii* | Art14 | Open Ocean, Halifax Canada | **JX437392** |  |
| *T. nordenskioeldii* | Art21 | Bedford Basin, Canada | **JX437393** |  |
| *T. nordenskioeldii* | Art24 | Bedford Basin, Canada | **JX437390** |  |
| *T. mala* Takano | **N/A** | Unknown | HM991693.1 |  |
| *T. oceanica* Hasle | IKETo062105 | Passamaquody Bay, Canada | **JX437375** | DITS364-08 |
| *T. oceanica* | N/A | Unknown | HM991696.1 |  |
| *T. oceanica* | **CCMP1001** | Atlantic, Continental Shelf | **JX437376** | DITS207-08 |
| *T. oestrupii v. venrickae* Fryxell & Hasle | **CC03-15** | Clam Creek, USA | DQ514870.1 |  |
| *T. cf pacifica* Gran & Angst | FB02-35 | San Francisco Bay, USA | DQ514888.1 |  |
| *T. pacifica* | **N/A** | Unknown | HM991697.1 |  |
| *T. proschkinae* Makarova | **IKETp091305** | Deadman's Harbor, Canada | **JX437372** | DITS365-08 |
| *T. pseudonana* Hasle & Heimdal | CCMP 1007 | Chincoteague, USA | DQ093367.1 |  |
| *T. pseudonana* | **CCMP1335** | Forge River, Long Island, USA | AY485452.1 |  |
| *T. pseudonana* | ETC1 | Lake Erie, USA | DQ514862.1 |  |
| *T. pseudonana* | NEPC709 | Hood Canal, USA | DQ514863.1 |  |
| *T. pseudonana* | CCMP1057 | Dabob Bay, USA | DQ514880.1 |  |
| *T. punctigera* (Castracane) Hasle | MHtp1 | North Sea | AJ810856.1 |  |
| *T. punctigera* | AWI | Unknown | AY485526.1 |  |
| *T. punctigera* | **FB02-06** | San Francisco Bay, USA | DQ514885.1 |  |
| *T. punctigera* | NB02-22 | Narragansett Bay, USA | DQ514893.1 |  |
| *T. ritscheri* (Hustedt) Hasle | **LC01-12** | Drake Passage, Southern Ocean | DQ514891.1 |  |
| *T. rotula* Meunier | CCMP1812 | Orcas Island, USA | **JX437379** | DITS149-08 |
| *T. rotula* | CCMP1647 | Bay of Naples, Italy | AF462058.1 |  |
| *T. rotula* | CCMP1018 | La Jolla, USA | AF462059.1 |  |
| *T. rotula* | **Art23** | Bedford Basin, Canada | **JX437380** |  |
| *T. rotula* | N/A | Unknown | X85397.1 |  |
| *Thalassiosira sp.1* | **CCMP1065** | Baffin Bay, Canada | DQ514881.1 |  |
| *Thalassiosira sp.1* | **CCMP1064** | Baffin Bay, Canada | EF192997.1 |  |
| *Thalassiosira sp.2 (as T. antarctica in GenBank****)*** † | **T1** | Barents Sea | EF140621.1 |  |
| *T. tenera* Proshkina-Lavrenko | MHtt1 | North Sea | AJ810858.1 |  |
| *T. tenera* | **N/A** | Unknown | HM991701.1 |  |
| *T. tumida* (Janisch) Hasle | CCMP1465 | Unknown | DQ093368.1 |  |
| *T. tumida* | **CCMP1469** | McMurdo Sound, Antarctica | DQ514883.1 |  |
| *T. tumida* | LA09-20 | Drake Passage, Southern Ocean | DQ514890.1 |  |
| *T. weissflogii* (Grunow) Fryxell & Hasle | CCMP1336 | Long Island, USA | **JX437395** | DITS155-08 |
| *T. weissflogii* | CCMP1587 | Jakarta, Indonesia | EF585582.1 |  |
| *T. weissflogii* | CCAP1085/1 | Gorleston-on-Sea, England | FJ600728.1 |  |
| *T. weissflogii* | N/A | Unknown | AF374477.2 |  |
| *T. weissflogii* | CCMP1049 | Amityville, USA | AY485445.1 |  |
| *T. weissflogii* | CCMP1010 | Gulf Stream, Atlantic | DQ514879.1 |  |
| *T. weissflogii* | CCMP1050 | Mar Slough, USA | GQ281043.1 |  |
| *T. weissflogii* | **CCMP1048** | East Falmouth, USA | GU594641.1 |  |
| *T. weissflogii* | p928 | Unknown | AJ535170.1 |  |
| *Skeletonema ardens* Sarno & Zingone | **CCMP794** | Singapore | DQ396520.1 |  |
| *S. ardens* | CS347 | Gulf of Carpentaria, Australia | DQ396521.1 |  |
| *S. japonicum* Zingone & Sarno | **CCMP1281** | Santa Cruz Island, USA | AY485473.1 |  |
| ***S. costatum* (Greville) Cleve** | **CCAP1077/4 A** | Strait of Georgia, Canada | AY684942.1 |  |
| *S. costatum* | CCAP1077/4 B | Strait of Georgia, Canada | AY684943.1 |  |
| *S. costatum* | CCAP1077/4 C | Strait of Georgia, Canada | AY684944.1 |  |
| *S. costatum* | CCAP1077/4 D | Strait of Georgia, Canada | AY684945.1 |  |
| *S. costatum* | CCAP1077/4 2A | Strait of Georgia, Canada | AY684946.1 |  |
| *S. costatum* | CCAP1077/4 2B | Strait of Georgia, Canada | AY684947.1 |  |
| *S. costatum* | CCAP1077/4 2C | Strait of Georgia, Canada | AY684948.1 |  |
| *S. costatum* | CCAP1077/4 2D | Strait of Georgia, Canada | AY684949.1 |  |
| *S. costatum* | NB02-45 2A | Narrangaset Bay, USA | AY684968.1 |  |
| *S. costatum* | NB02-45 A | Narrangaset Bay, USA | AY684969.1 |  |
| *S. costatum* | NB02-45 2B | Narrangaset Bay, USA | AY684970.1 |  |
| ***S. costatum*** | **Art31** | West Coast Port WO78 | **JX437370** |  |
| *S. dohrnii* Sarno & Kooistra | **SZN-B104** | Gulf of Naples, Italy | AJ632210.1 |  |
| *S. dohrnii* | SZN-B105 | Gulf of Naples, Italy | AJ632211.1 |  |
| *S. grethae* Zingone & Sarno | N/A | Unkown | X85395.1 |  |
| *S. grethae* | CCAP1077-3 | Narragansett Bay, USA | AJ632204.1 |  |
| *S. grethae* | **CCMP780** | Wood's Hole, USA | AJ632205.1 |  |
| *S. grevilleii* Zingone & Sarno | **CCMP1685** | Muscat, Oman | DQ396512.1 |  |
| *S. marinoi* Zingone & Sarno | CCMP789 | Unknown | **JX437369** | DITS327-08 |
| *S. marinoi* | N/A | Unknown | AF462060.1 |  |
| *S. marinoi* | CCMP1009 | Continental Slope, Atlantic | AJ535165.1 |  |
| *S. marinoi* | SZN-B121 | North Adriatic | AJ632212.1 |  |
| *S. marinoi* | SZN-B146 | North Adriatic | AJ632213.1 |  |
| *S. marinoi* | SZN-B118 | North Adriatic | AJ632214.1 |  |
| *S. marinoi* | **SZN-B120** | North Adriatic | AJ632216.1 |  |
| *S. marinoi* | MMDL5613 | China Sea, China | EF138931.1 |  |
| *S. marinoi* | MMDL5615 | China Sea, China | EF138932.1 |  |
| *S. marinoi* | MMDL5616 | China Sea, China | EF138933.1 |  |
| *S. marinoi* | MMDL5617 | China Sea, China | EF138934.1 |  |
| *S. marinoi* | MMDL5621 | China Sea, China | EF138936.1 |  |
| *S. marinoi* | MMDL5623 | China Sea, China | EF138937.1 |  |
| *S. marinoi* | MMDL5625 | China Sea, China | EF138939.1 |  |
| *S. marinoi* | MMDL5628 | China Sea, China | EF138940.1 |  |
| *S. marinoi* | CCMP781 | Fladenground, North Sea | EF433519.1 |  |
| *S. marinoi* | K669 | Unknown | EF433521.1 |  |
| *S. marinoi* | 090520M | Sweden | HM236345.1 |  |
| *S. marinoi* | 090602X | Sweden | HM236346.1 |  |
| *S. marinoi* | ST13 | Sweden | HM236347.1 |  |
| *S. marinoi* | V8 | Sweden | HM236348.1 |  |
| *S. marinoi* | V52 | Sweden | HM236349.1 |  |
| *S. menzelii* | SZN-B82 | Gulf of Naples, Italy | AJ632217.1 |  |
| *S. menzelii* | SZN-B83 | Gulf of Naples, Italy | AJ632218.1 |  |
| *S. pseudocostatum* Medlin | SZN-B77 | Gulf of Naples, Italy | AJ632207.1 |  |
| *S. pseudocostatum* | SZN-B78 | Gulf of Naples, Italy | AJ632208.1 |  |
| *S. pseudocostatum* | SZN-B80 | Gulf of Naples, Italy | AJ632209.1 |  |
| *S. pseudocostatum* | CCAP1077/7 | Alexandria, Egypt | AY684950.1 |  |
| *S. pseudocostatum* | CCAP1077/7 A | Alexandria, Egypt | AY684951.1 |  |
| *S. pseudocostatum* | CCAP1077/7 B | Alexandria, Egypt | AY684952.1 |  |
| *S. pseudocostatum* | **CCAP1077/7 C** | Alexandria, Egypt | AY684953.1 |  |
| *S. pseudocostatum* | CCAP1077/7 D | Alexandria, Egypt | AY684954.1 |  |
| *S. pseudocostatum* | CCAP1077/7 E | Alexandria, Egypt | AY684955.1 |  |
| *S. pseudocostatum* | CCAP1077/7 G | Alexandria, Egypt | AY684956.1 |  |
| *S. pseudocostatum* | CCAP1077/7 H | Alexandria, Egypt | AY684957.1 |  |
| *S. pseudocostatum* | CCAP1077/7 I | Alexandria, Egypt | AY684958.1 |  |
| *S. pseudocostatum* | CCAP1077/6 | Australia, South Pacific Ocean | X85393.1 |  |
| *S. subsalsum* (Cleve-Euler) Bethge | CCAP1077/8 | Lower Lough Erne, Ireland | AY485494.1 |  |
| *S. subsalsum* | **CCAP1077/8 A** | Lower Lough Erne, Ireland | AY684959.1 |  |
| *S. subsalsum* | CCAP1077/8 B | Lower Lough Erne, Ireland | AY684960.1 |  |
| *S. subsalsum* | CCAP1077/8 C | Lower Lough Erne, Ireland | AY684961.1 |  |
| *S. subsalsum* | CCAP1077/8 D | Lower Lough Erne, Ireland | AY684962.1 |  |
| *S. subsalsum* | CCAP1077/8 E | Lower Lough Erne, Ireland | AY684963.1 |  |
| *S. subsalsum* | CCAP1077/8 F | Lower Lough Erne, Ireland | AY684964.1 |  |
| *S. subsalsum* | CCAP1077/8 G | Lower Lough Erne, Ireland | AY684965.1 |  |
| *S. subsalsum* | CCAP1077/8 H | Lower Lough Erne, Ireland | AY684966.1 |  |
| *S. subsalsum* | CCAP1077/8 J | Lower Lough Erne, Ireland | AY684967.1 |  |
| *S. subsalsum* | MMDL5619 | China Sea, China | EF138935.1 |  |
| *S. tropicum* Cleve | **M98** | Italy | DQ396513.1 |  |
| *S. tropicum* | M99 | Italy | DQ396514.1 |  |
| *S. tropicum* | B205 | Montevideo, Uruguay | DQ396515.1 |  |
| *S. tropicum* | B210 | Montevideo, Uruguay | DQ396516.1 |  |
| *S. tropicum* | CCMP2070 | Gulf of Panama | DQ396517.1 |  |
| **Cymatosirales** | | | |  |
| *Arcocellulus mammifer* Hasle, von Stosch & Syvertsen | **CCMP132** | Unknown | **JX437407** | DITS189-08 |
| *Brockmanniella brockmannii* (Hustedt) Hasle, von Stosch & Syvertsen | CCMP151 | Unknown | **JX437414** | DITS188-08 |
| *B. brockmannii* | **PC1A3** | Wood's Point, NB, Canada | **JX437415** | VFOUR002-12 |
| *B. brockmannii* | PC1B2 | Wood's Point, NB, Canada | **JX437416** | VFOUR003-12 |
| *B. brockmannii* | PC1B3 | Wood's Point, NB, Canada | **JX437417** | VFOUR004-12 |
| *B. brockmannii* | PC1C2 | Wood's Point, NB, Canada | **JX437418** | VFOUR005-12 |
| *Campylosira cymbelliformis* (Schmidt) Grunow ex Van Heurck | **JMRB4** | Dorchester, NB, Canada | **JX437411** | VFOUR006-12 |
| *C. cymbelliformis* | WCC10.5 | Sentson Beach, USA | **JX437412** | VFOUR007-12 |
| *C. cymbelliformis* | CCC-1 | Corpus Christy Bay, USA | HQ912623.1 |  |
| *Cymatosira belgica* (Grunow) | **CCMP345** | Unknown | **JX437413** | VFOUR008-12 |
| *Extubocellulus spinifer* (Hargreaves & Guillard) Hasle, Stosch & Syvertsen | **CCMP393** | Falmouth, USA | **JX437409** | DITS176-08 |
| *E. cribriger* Hasle, Stosch & Syvertsen | **CCMP391** | Unknown | **JX437410** | DITS177-08 |
| *Minutocellus polymorphus* (Hargraves & Guillard) Hasle, Stosch, & Syvertsen | CCMP499 | Sandy Hook, USA | **JX437404** | DITS170-08 |
| *M. polymorphus* | **CCMP501** | Great Salt Bay, USA | **JX437405** | VFOUR036-12 |
| *M. polymorphus* | CCMP1701 | Gulf of Oman, Arabian Sea | **JX437406** | DRBAR001-11 |
| *Papiliocellulus elegans* Hasle, von Stosch & Syvertsen | **CCMP3125** | Wilmington, USA | **JX437408** | VFOUR011-12 |
| *Plagiogrammopsis vanheurckii* (Grunow) Hasle, von Stosch & Syvertsen | BSA3 | Sackville, NB, Canada | **JX437419** | VFOUR012-12 |
| *P. vanheurckii* | **JMRA1** | Dorchester, NB, Canada | **JX437420** | VFOUR013-12 |
| *P. vanheurckii* | BSB3 | Sackville, NB, Canada | **JX437421** | VFOUR014-12 |
| *P. vanheurckii* | BSB4 | Sackville, NB, Canada | **JX437422** | VFOUR015-12 |
| *P. vanheurckii* | BSC1 | Sackville, NB, Canada | **JX437423** | VFOUR016-12 |
| *P. vanheurckii* | JMRA2 | Dorchester, NB, Canada | **JX437424** | VFOUR017-12 |
| *P. vanheurckii* | PC1A4 | Wood's Point, NB, Canada | **JX437425** | VFOUR018-12 |
| *P. vanheurckii* | PC1B1 | Wood's Point, NB, Canada | **JX437426** | VFOUR019-12 |
| *P. vanheurckii* | PC2A1 | Wood's Point, NB, Canada | **JX437427** | VFOUR020-12 |
| *P. vanheurckii* | PC2A3 | Wood's Point, NB, Canada | **JX437428** | VFOUR021-12 |
| *P. vanheurckii* | PC2A4 | Wood's Point, NB, Canada | **JX437429** | VFOUR022-12 |
| *P. vanheurckii* | PC2B1 | Wood's Point, NB, Canada | **JX437430** | VFOUR023-12 |
| *P. vanheurckii* | PC2B2 | Wood's Point, NB, Canada | **JX437431** | VFOUR024-12 |
| *P. vanheurckii* | PC2B4 | Wood's Point, NB, Canada | **JX437432** | VFOUR025-12 |
| *P. vanheurckii* | PC2C1 | Wood's Point, NB, Canada | **JX437433** | VFOUR026-12 |
| *P. vanheurckii* | PC2C2 | Wood's Point, NB, Canada | **JX437434** | VFOUR027-12 |
| **Fragilariales** | | | |  |
| *Tabularia fasiculata* (C.Agardh) Williams & Round | 308AC | Cape Tormentine, NB, Canada | **JX437441** | VFOUR028-12 |
| *T. fasciculata* | 308AG | Cape Tormentine, NB, Canada | **JX437442** | VFOUR029-12 |
| *T. fasciculata* | 823A | Cape Tormentine, NB, Canada | **JX437443** | VFOUR030-12 |
| *T. fasciculata* | 502A | St Andrew's, NB, Canada | **JX437444** | VFOUR031-12 |
| *T. fasciculata* | 206A UK | Ukraine | **JX437445** | VFOUR032-12 |
| *T. fasciculata* | 206C UK | Ukraine | **JX437446** | DITS352-08 |
| *T. fasciculata* | 206H UK | Ukraine | **JX437447** | DITS350-08 |
| *T. fasciculata* | 615A | Vancouver, Canada | **JX437448** | VFOUR033-12 |
| *T. fasciculata* | 615B | Vancouver, Canada | **JX437449** | VFOUR034-12 |
| *T. fasciculata* | 408A | Nanaimo Bay, Canada | **JX437450** | VFOUR035-12 |
